# COVID-19–sensitive tumour response: 2-year assessment of the SARS-CoV-2 humoral response in cancer patients in oncology hospital in Poland

**DOI:** 10.1007/s00262-024-03895-z

**Published:** 2024-12-21

**Authors:** Piotr Kosiorek, Bożena Mikołuć, Samuel Stróż, Anna Hryniewicz, Dorota E. Kazberuk, Robert Milewski, Anna Grzeszczuk, Magdalena J. Borkowska, Anna Stasiak‐Barmuta

**Affiliations:** 1 Department of Emergency, Maria Sklodowska‐Curie Bialystok Oncology Centre, Białystok, Poland; 2 Department of Clinical Immunology, Medical University of Białystok, Białystok, Poland; 3 Department of Pediatrics, Rheumatology, Immunology and Metabolic Bone Diseases, Bialystok, Poland; 4 Department of Rehabilitation, Medical University of Białystok, Białystok, Poland; 5 Department of Radiotherapy, Maria Sklodowska‐Curie Bialystok Oncology Centre, Białystok, Poland; 6 Department of Biostatistics and Medical Informatics, Medical University of Białystok, Białystok, Poland; 7 Department of Infectious Diseases and Neuroinfection, Medical University of Białystok, Białystok, Poland

**Keywords:** Breast cancer, Lung cancer, Colon cancer, SARS-CoV-2, COVID-19

## Abstract

**Abstract:**

Vaccination has been considered the most crucial defence against viral infections, including SARS-CoV-2. Numerous reports have demonstrated the effectiveness of the above vaccines in oncological patients. It has also been proven that, apart from vaccinations and oncological therapy, the course of the cancer process itself influences the magnitude of the humoral response, especially in people after infection with SARS-CoV-2. The phenomenon we observe seems to confirm the presence of a "natural" defence potential in a cancer patient's body, in this case, directed against infection with a viral pathogen. A "stronger" antiviral response also explains the asymptomatic course of SARS-CoV-2 infection in some of the above patients. To what extent the SARS-CoV-2 infection weakened the "natural" potential of the anticancer response in these patients remains an open question.

**Objective:**

This study aimed to answer the question about the impact of the cancer process on the humoral response in oncological patients vaccinated against SARS-CoV-2 infection and in patients after COVID-19.

**Material and methods:**

One thousand six hundred and sixty-eight people were observed. Over 2 years, 5,082 SARS-CoV-2 IgG and IgM antibody samples were determined. The concentration of antibodies was assessed in groups of oncological patients: those undergoing anticancer therapy after contracting COVID-19 and those after vaccination against the SARS-CoV-2 infection.

**Results:**

The obtained results indicate a naturally more significant humoral response in oncological patients who have not been vaccinated and have not undergone anticancer therapy, such as radiotherapy, chemotherapy, or surgical intervention. The above observation applies to patients with breast, lung, colon, kidney, and testicular cancer, although the response varies significantly depending on the type of cancer.

**Graphical abstract:**

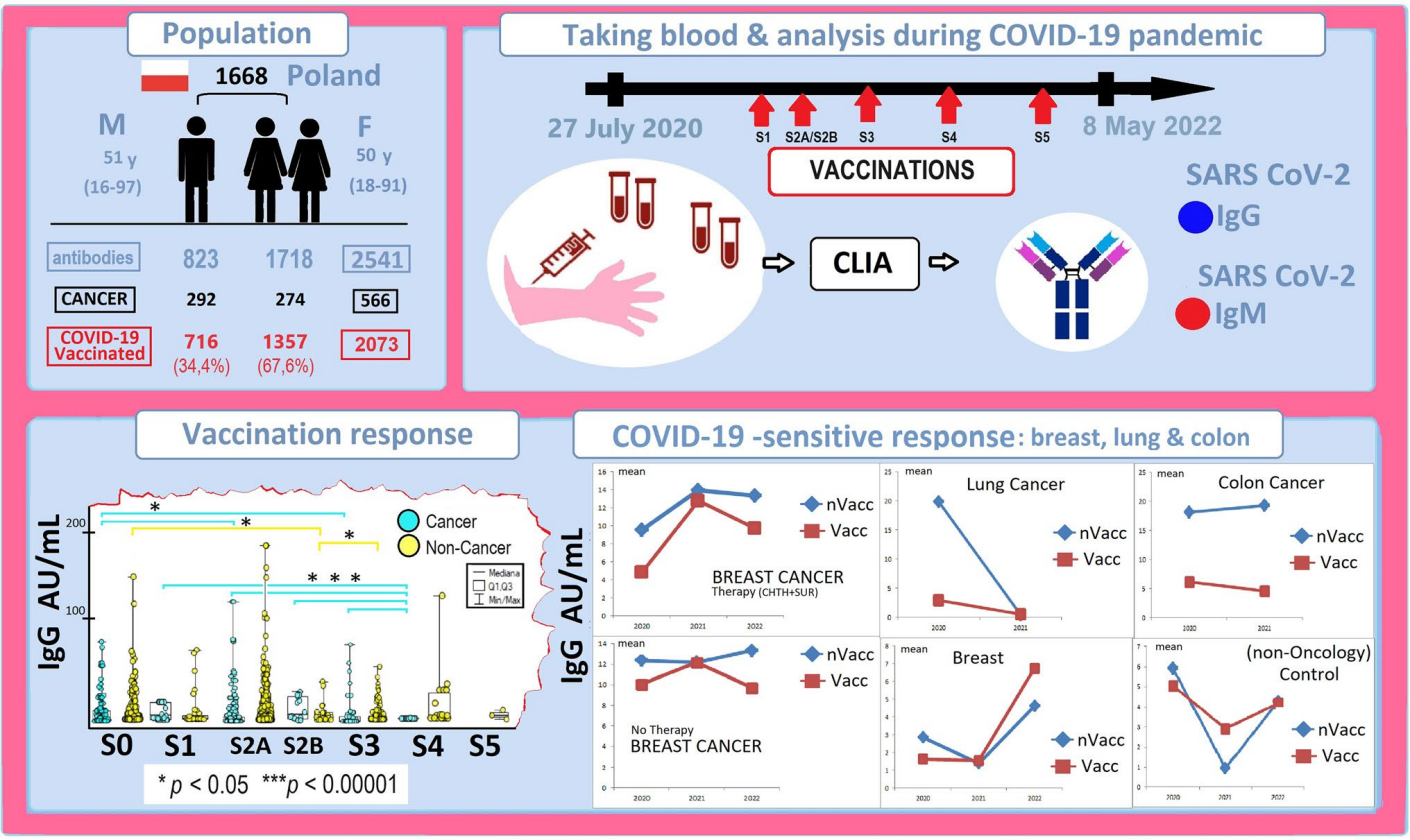

## Introduction

Immune response to beta-coronavirus (SARS-CoV-2; severe acute respiratory syndrome coronavirus 2) does not always lead to the development of a clinical syndrome called COVID‐19 (coronavirus disease 2019), among others, due to the low response of antibodies in the cancer population [1, 2]. The prevalence of asymptomatic SARS-CoV-2 infection in patients with cancer is similar to the general population, while at the same time, patients with cancer may be at increased risk of severe course of COVID-19 [3]. The phenomenon of asymptomatic infection is believed to include, among others, the inflammation that accompanies every cancer disease, involving components of innate and acquired immunity [4]. In the literature, we find data that only every third patient with diagnosed cancer undergoing anticancer therapy responds to the complete two-dose vaccination by producing antibodies [5]. The booster effect (third dose) equalizes the chances between patients' subpopulations during oncological treatment [6, 7].

It is worth noting that during a 12-month observation, it was proven that chemotherapy or radiotherapy increases the pool of immunoglobulin class G (IgG) antibodies against SARS-CoV-2 in cancer patients [8]. It has also been proven that documenting SARS-CoV-2 infection with a molecular test is insufficient if it does not correlate with the patient's clinical condition [9]. Solodky et al*.* show that oncological treatment 1 month after identifying the virus makes it undetectable in tests [10]. Hence, an essential message for cancer patients is to get vaccinated, regardless of the test results, which may be false negative or positive.

As it has been shown, the imperfection of diagnostic tests, primarily their sensitivity, influences the virus to remain below the detection value in the laboratory; the clinical characteristics of such a situation are called long-COVID-19, a disease in which the shedding of the virus is also prolonged as the disease drags on.

Oncology patients suffer from a chronic inflammatory process caused by the presence of cancer cells. This process involves innate and acquired immunity, gradually depleting the body's defence reserves. Therefore, only chemotherapy and radiotherapy, which deprive the body of the source or cause of chronic inflammation, can stimulate CD8 + T cells, B lymphocytes and plasma cells, and immune memory cells to an appropriate humoral response [8].

Only the third dose of the vaccine against SARS-CoV-2 infection works similarly in patients with soft tissue tumours [11]. This phenomenon has not been observed in oncohematological patients suffering from B-cell leukaemias or lymphomas, where as a result of permanent myelosuppression, the humoral response is impaired [8]. The exception is patients suffering from Hodgkin's lymphoma, where antibody-dependent SARS-CoV-2 induction of anti-tumour response [12], and patients with multiple myeloma [9]. In the case of non-haematological cancers, such as breast cancer in women and prostate cancer, studies conducted on small groups of patients showed an increased humoral response, which was associated with chronic inflammation and immunosuppressive treatment [11].

This study aimed to answer the question about the impact of the cancer process on the humoral response in oncological patients vaccinated against SARS-CoV-2 infection and in patients after COVID-19. In the conducted studies, attention was paid to the potential impact of the anti-infective response, in this case, the antiviral response, on the potential of the anticancer response. This is important because a post-infectious or post-vaccination increase in the concentration of IgG antibodies, secondary response antibodies, provides a chance to "strengthen" the anti-infective humoral response for many months.

## Material and methods

### Patients and protocol

The medical records of patients hospitalized in the Oncology Hospital in Poland were subjected to a retrospective analysis from 27 July 2020 to 8 May 2022. The study involved 1668 patients (1718 females and 823 males). Each patient had blood taken for antibody tests by a doctor's referral during a visit to the office. Oncology patients were not selected or assigned to selected groups. No protocol was maintained for the reasons for antibody referrals. The indication for antibody testing was solely the desire to learn about humoral immunity before or after the vaccination of an oncology patient, for the patient's knowledge and the doctor's. It did not concern the assessment of the treatment administered to a given group of patients, vaccinated or unvaccinated. The patient's data did not include inflammation or comorbidities. The clinical condition of the patient, taking into account specific biochemical parameters, determines the initiation of oncological treatment. Chronic inflammation in oncological treatment is typical in the biochemical picture of these patients. No correlation of clinical and biochemical data was planned. As mentioned, oncological treatment concerned patients without exacerbations of the underlying disease, including organ complications: renal, cardiac, and neurological, etc., who were treated in departments of other hospitals.

The natural distribution of humoral responses in populations depends on age, gender, diseases and treatment processes, and current health status. Vaccinations were not performed if the patient was a carrier of the SARS-COV-2 virus, had biological material, and was likely to be infected. According to epidemiological guidelines, people were vaccinated > 28 days after a positive molecular SARS-CoV-2 test and > 14 days after the cessation of symptoms of the disease. Patients in clinical severe conditions did not undergo oncological treatment.

A group of people with a histopathologically confirmed diagnosis of oncological disease (Cancer), people undergoing oncological diagnosis (N-Cancer), and a group of control people, hospital employees tested before and after vaccination (Non-oncology), were distinguished. The groups were not randomized. Additionally, a group was distinguished by diagnoses of autoimmune diseases (autoimmunology) and specific diseases (O-cancer and H-cancer).

Because the wave of SARS-CoV-2 infections repeatedly affected our population, we did not keep statistics on flu-like illnesses. COVID-19 molecular and antigen tests were performed routinely when severe infections were suspected. There was no purpose in selecting cancer patients with or without vaccination. Statistics analysis and groups S1-S5 were specified retrospectively after the pandemic.

All of them were referred for antibody tests voluntarily due to their history of diseases, lack of immunity, and the possibility of receiving vaccinations during oncological treatment (chemotherapy, radiotherapy, and surgery). Some of them had antibodies ordered several times. Medical staff were required to be fully vaccinated against COVID-19 during the pandemic. Because the government regulated vaccine administration in 2021, the first group was medical personnel, followed by people over 60, and only then cancer patients. The first administration of the vaccine was mainly with Pfizer (BNT162b2; Comirnaty), AstraZeneca (AZD1222; Vaxzevria), Moderna (MRNA-1273; Spikevax), and Johnson&Johnson (Ad26.COV2.S). Booster doses (2021/2022) were often the Pfizer or Moderna vaccine.

In our study, 82% of the population was vaccinated, including 416 samples from cancer patients who received only the Pfizer-BioNTech BNT162b2 vaccine (Comirnaty). BNT162b2 is 95% effective in preventing COVID-19, and similar vaccine efficacy is observed across subgroups defined by age, sex, race, and ethnicity. The Bioethics Commission of Medical University approved our research.

### IgG and IgM measurement

Our study materials were blood specimens collected through venipuncture sampling. The concentration of antibodies was evaluated four hours after blood collection. If an immediate assessment was not possible, the serum was collected and stored at − 80 ◦C.

During 2-year observation, two class antibodies were detected (*n* = 5082). The levels of IgM and IgG antibodies (*n* = 2541 each), oriented specifically towards SARS-CoV-2, which were detected by chemiluminescent immunoassay (CLIA; MAGLUMI, Snibe Diagnostic, Shenzhen, China). Results greater than or equal to 0.2 AU/mL SARS-CoV-2 IgG and 1.0 AU/mL IgM were considered indicative of a reaction and regarded as positive, according to the manufacturer’s protocol.

### Statistical analysis

Non-parametric statistical methods were applied for the case–control analyses because many of the considered variables were not normally distributed. The Mann–Whitney test was adopted to compare two groups to each other, whereas the Kruskal–Wallis, with the post-hoc test, was applied to assess more than two groups. Results were considered statistically significant when p < 0.05. We used PQStat Software 2021 (PQStat Software, Poznan, Poland) and Tibco Statistics 13.3 (TIBCO Software Inc., Palo Alto, CA, USA) for statistical analyses.

## Results

Table 1 contains the characteristics of the study, and Table 2 is supplemented with units of diseases examined and the share of vaccinated people.

**Table 1:** Characteristics of study

*n* (%) of total antibodies mesurements*	Quantity (all)	Vaccinated	2020	2021	2022
			Quantity	Quantity	Quantity
All	2541 (100%)*	2073 (100%)	606	1361	574
Man	823 (32.39)	716 (34.54)	124	498	201
Woman	1718 (67.61)	1357 (65.46)	482	863	373
Clinical division					
Oncology group	1714 (67.44)	1369 (66.04)	400	1007	307
Cancer group	604 (23.77)	498 (24.02)	65	455	84
Cancer	566 (22.27)	465 (22.43)	63	430	73
O-Cancer	29 (1.14)	24 (1.16)	2	18	9
H-Cancer	9 (0.35)	9 (0.43)	0	7	2
Non-Cancer group					
Autoimmunology	104 (4.09)	73 (3.52)	33	50	21
Non-Cancer	1006 (39.59)	798 (38.49)	302	502	202
Non-Oncology group					
	827 (32.56)	704 (33.96)	206	354	267
Treatment division	599 (23.58)	494 (23.83)	62	453	84
Radiotherapy	376 (22.54)	281 (13.56)	38	287	51
+ chemotherapy	327 (12.87)	232 (11.19)	37	261	29
Chemotherapy	435 (26.08)	316 (15.24)	42	344	49
+ surgery	305 (12.00)	204 (9.84)	41	232	32
Surgery	446 (26.74)	326 (15.72)	59	337	50
+ radiotherapy	283 (11.14)	194 (9.36)	39	213	31
Non-treatment					
	1942 (76.47)	1579 (81.31)	544	908	490
		Cancer	2020	2021	2022
Vaccinated division	2073 (100%)	566 (100%)	499	1089	484
Pfizer	1835 (88.52)	416 (73.50)	486	985	364
Moderna	67 (3.23)	25 (4.42)	4	45	18
AstraZeneca	98 (4.72)	31 (5.48)	6	39	53
Johnson&Johnson	75 (3.62)	25 (4.42)	5	21	49
Non-vaccinated					
	469	107	107	272	90
Sex	median y (min–max)	Cancer 566 (100%)	median y (min–max)	median y (min–max)	median y (min–max)
Man	51 (16–97)	292 (51.59)	51(21–86)	57 (16–97)	44 (21–84)
Woman	50 (18–91)	274 (48.41)	48 (21–80)	53 (18–91)	48 (22–88)
Age range					
< 40	34 (16–39)	28 (4.95)	34(21–39)	34(16–39)	35(21–39)
40–60	50 (41–59)	135 (23.85)	52(41–59)	50(41–59)	49(41–59)
> 60	70 (61–97)	364 (64.31)	65(61–86)	70.5(61–97)	68(61–88)

*All antibody measurements, SARS-CoV-2 IgG and IgM (each *n* = 2541). All antibody concentration results are in AU/mL. The population of study participants was normally distributed. The cancer group (Cancer, O-Cancer, H-Cancer) was concerned only with histopathologically diagnosed neoplasms with treatment (including palliative treatment): radiotherapy, chemotherapy, and surgery. We excluded some cancers (O-Cancer) with different origins, while H-Cancer is the specific Hodgkin lymphoma. Control, non-treatment persons were hospital staff

**Table 2:** Detailed division of the conducted research (disease entity, (*n*) number of tests (%), and number of samples from vaccinated people (Vacc)

Cancer	*n* = 566 (%)	Vacc *n* = 429	*p*-Value	N-Cancer	*n* = 1006 (%)	Vacc *n* = 818	*p*-Value
Breast	119 (21.03)	72	*p* = 0.009*	Breast	259 (25.75)	206	*p* = *0.149*
Prostate	88 (15.55)	79	*p* = *0.101*	Skin	110 (10.93)	81	*p* = *0.983*
	75 (13.25)	45	*P* = 0.0001*	Polyps intestine	71 (7.06)	56	*p* = *0.191*
Lung Colon	52 (9.20)	39	*p* = *0.068*	Spine	63 (6.26)	56	*p* = *0.392*
Tongue	39 (6.89)	37		Intestine	60 (5.96)	58	*p* = *0.167*
Rectal	33 (5.83)	28	*p* = *0.761*	Breast lumps	54 (5.37)	48	*p* = *0.679*
Skin	28 (4.95)	27		Thyroid nodule	32 (3.18)	25	
Kidney	27 (4.77)	20	*p* = 0.007*	Thyroid	31 (3.08)	21	*p* = *0.359*
Uterine	25 (4.41)	17	*p* = *0.169*	Gastritis	30 (2.98)	20	*p* = *0.38*
Brain	19 (3.36)	19		Hypothyroidism	29 (2.88)	22	
Testical	10 (1.77)	7	*p* = 0.005*	Ovarian	23 (2.29)	21	*p* = *0.155*
Stomach	9 (1.59)	7		Lung	23 (2.29)	21	*p* = *0.411*
Oesophageal	7 (1.24)	7		Naevus	22 (2.19)	17	*p* = *0.968*
Thyroid	2 (1.06)	4		Stomach	20 (1.99)	18	*p* = *0.377*
Pancreas	5 (0.88)	2	*p* = *0.386*	Ovarian cyst	20 (1.99)	13	*p* = *0.342*
Glioblastoma	3 (0.53)	3		Prostate hyperplasia	18 (1.79)	12	*p* = *0.882*
Laryngeal	3 (0.53)	3		Diverticulitis	15 (1.49)	13	*p* = *0.923*
Gallblader	3 (0.53)	3		Naevus pigmentosus	14 (1.39)	12	*p* = *0.059*
Bladder	3 (0.53)	3		Lipoma	13 (1.29)	4	*p* = *0.392*
Sarcoma	2 (0.35)	2		Nephrolithiasis	12 (1.19)	11	
Vulvar	2 (0.35)	2		Seborrheic papilloma	10 (1.00)	5	
Thymus	2 (0.35)	2		Uterine	8 (0.80)	6	*p* = *0.092*
Liver	2 (0.35)	2		Struma nodosa	7 (0.70)	6	
Bone	2 (0.35)	2		Fibroma	7 (0.70)	4	*p* = 0.05*
Pleural mesothelioma	1 (0.18)	1		Endometriosis	7 (0.70)	4	*p* = *0.582*
Carcinoid tumour	1 (0.18)	1		Uterine polyps	5 (0.50)	3	*p* = *0.767*
Salivary gland	1 (0.18)	1		Pancreatic cyst	4 (0.40)	3	
Ovarian	1 (0.18)	1		Hernia	4 (0.40)	2	
Cervical	1 (0.18)	1		Common warts	3 (0.30)	2	
Astrocytoma	1 (0.18)	1		Sciatica	3 (0.30)	1	
		Vacc *n* = 23		Prostatitis	3 (0.30)	1	
O-Cancer	*n* = 29 (%)		*p-Value*	Uterine fibrosis	3 (0.30)	0	
Cervical dysplasia	11 (37.93)	11		Cervical	3 (0.30)	0	
Melanocytoma	7 (24.14)	6		Abscesses	3 (0.30)	3	
Myeloma	4 (13.79)	1		Gastric polyps	2 (0.20)	22	
Acustic neuroma	2 (6.90)	0		Nevus flammeus	2 (0.20)	10	
Lymphoma	2 (6.90)	2		Neuralgia	2 (0.20)	1	
Adenomyosis uterus	1 (3.45)	1		Kidney	2 (0.20)	2	
Thrombocythemia	1 (3.45)	1		Tinea corporis	1(0.10)	1	
Myeloid leukaemia	1 (3.45)	1		Testicular nodule	1 (0.10)	0	*p* = *0.304*
		Vacc *n* = 72		Pancreatic	1 (0.10)	1	
Autoimmunology	*n* = 104 (%)		*p-Value*	Ostitis	1 (0.10)	1	
Allergic disease	27 (25.96)	15	*p* = *0.286*	Nose nodule	1 (0.10)	1	
Rheumatoid arthritis	26 (25.00)	16	*p* = 0.006*	Liver	1 (0.10)	1	
Hashimoto`s disease	19 (18.27)	16	*p* = *0.240*	Herpes simplex	1 (0.10)	1	
Graves` disease	11 (10.58)	5	*p* = 0.004*	COPD #	1 (0.10)	1	
Hyperthyroidism	5 (4.81)	5		Fatty liver	1 (0.10)	1	
Sclerodermia	4 (3.85)	4					
Psoriasis	2 (1.92)	2		H-Cancer	*n* = 9 (%)	Vacc	*p-Value*
Lyme disease	2 (1.92)	2		Hodgkin`s lymphoma	9 (100)	9	
Liver haemangiomas	2 (1.92)	2					
Ulcerative colitis	2 (1.92)	2		Non-Oncology (Control)			
Autoimmune thyroiditis	2 (1.92)	2			*n* = 827(%)	Vacc	*p-Value*
Crohn`s disease	1 (0.96)	1			827 (100)	692	
Celiac disease	1 (0.96)	1					

^#^ COPD–Chronic Obstructive Pulmonary Disease; The cancer group had histopathologically confirmed disease diagnoses – N-cancer group undergoing oncological diagnosis. Patients undergoing oncological treatment during the SARS-CoV-2 pandemic did not show typical symptoms of COVID-19; the tests performed routinely for SARS-CoV-2 were primarily negative (PCR and antigen). Most patients had COVID-19 in 2020, vaccinations began in January 2021, and most had multiple scanty symptoms all the time. There was no severe COVID-19 in the observation group. Only 28 people died from carcinoma: brain, colon, lungs, pancreas, prostate, rectum, and uteri, but only six were vaccinated. This study is most relevant for Delta virus variants in Poland. We were unable to determine the type of virus variants in our laboratory. *p-Value (only available) between groups vaccinated (Vacc) and non-vaccinated

**Fig. 1:**
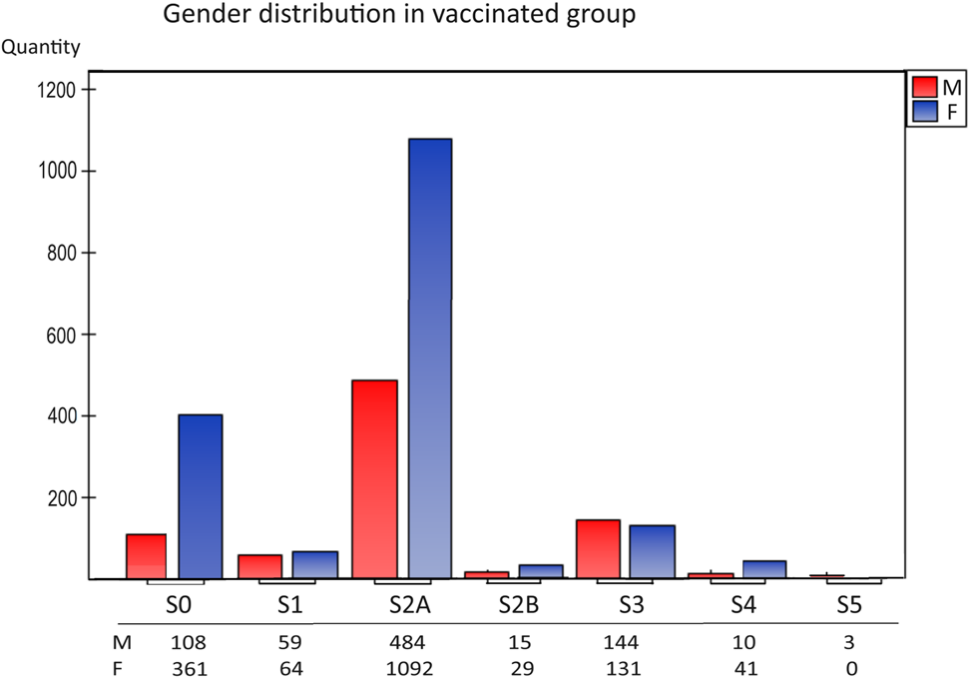
Characteristics of gender distribution in the vaccinated group (S1–S5). The number of sample representatives of each group is below the group symbol. There were no differences between sexes in groups—the S0-control group. S0 (no vaccination), S1 (single vaccine: Johnson&Johnson (J&J), AstraZeneca(AZ), Moderna, Pfizer (COM; Comirnaty), S2A (full two-dose one vaccination), and S2B (two-dose vaccination Pfizer and Moderna), S3, S4, S5-additional vaccination

The analysis of the distribution of vaccinations used (S1-S5) in our study showed that the most significant population were people fully vaccinated with BNT162b2, two-dose series mRNA vaccines dedicated to hospital workers and oncology patients 2021 (Fig. 1, group S2A). The next group consisted of people vaccinated with three doses (Fig. 1, group S3). Young people chose one-dose vaccination (S1), while few people were vaccinated with other preparations (mRNA-1273 vaccine (Moderna), Ad26.COV2.S vaccine (Johnson&Johnson) and AZD1222 vaccine (AstraZeneca), two-full dose BNT162b2 (S2A), two-full dose currently available vaccines (S2B), and received the fourth (S4) and fifth doses (S5).

**Fig. 2:**
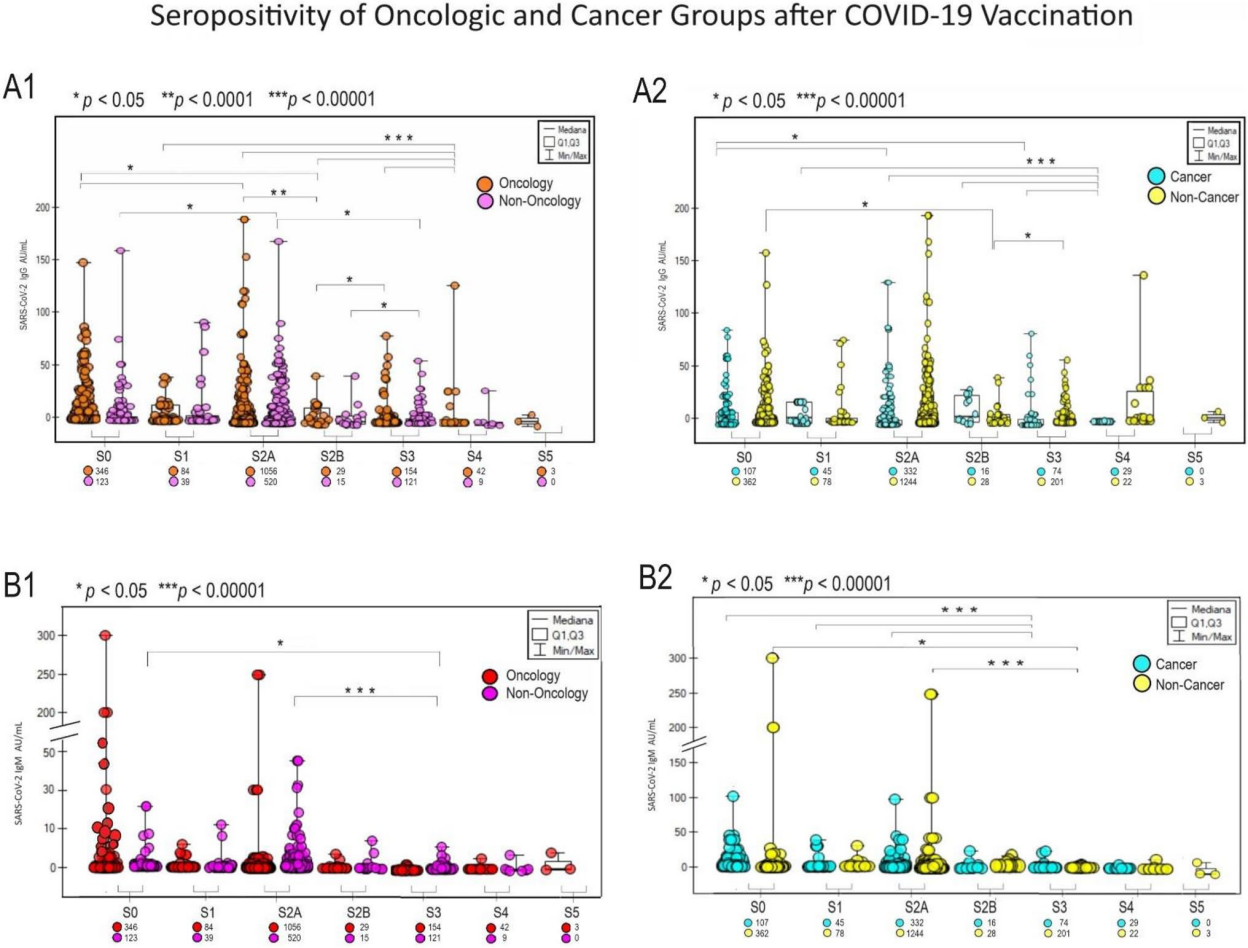
The diagrams present the significance of different SARS-CoV-2 IgG (**A1**) and IgM (**B1**) responses to COVID-19 vaccination between the oncologic versus non-oncologic group (A1, B1) and the cancer versus non-cancer Group (**A2**, **B2**). Mediana and correlation (p-value) between groups presented. Below the group symbol is the amounts of representatives of each group. The vaccinated group: S0 (no vaccination), S1 (single vaccine: Johnson&Johnson, AstraZeneca, Moderna, Pfizer), S2A (full two-dose one vaccination), and S2B (two-dose vaccination Pfizer and Moderna), S3, S4, S5 booster mostly Pfizer and Moderna. Antibody concentrations are measured in artificial units per mL (AU/mL). P-values represent comparison with groups (*) *p* < 0.05, (**) *p* < 0.001, (***) *p* < 0.0001

A significant response to vaccination was observed in the patient population oncology and cancer among unvaccinated people (Fig. 2 graphs A and B, for IgG and IgM appropriately). The highest IgG response occurred in the S3 group vaccinated oncology and cancer patients (*p* < 0.05) and S4 non-oncological and non-cancer (p < 0.00001) Fig. 2A1 and A2

A detailed breakdown of the cancer group into the most common types (Table 2) revealed differences between breast, lung, colon, kidney, and testicular cancer populations from other cancer and disease states in the COVID-19 pandemic (p-value significant).

### Breast *cancer*

**Fig. 3:**
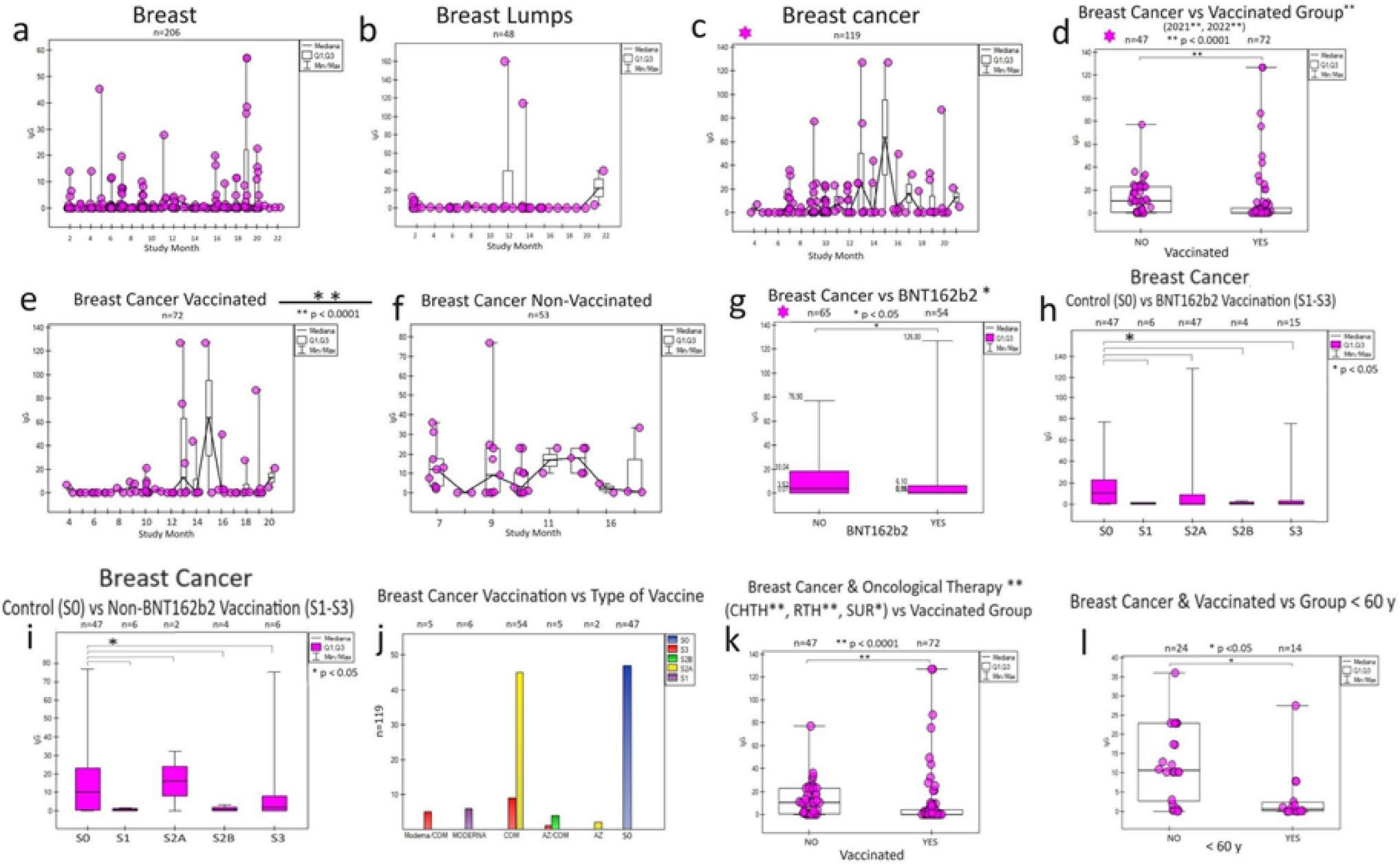
Specific humoral response of particular IgG to SARS-CoV-2 infection and COVID-19 vaccination in oncological patients with breast disease over 2 years (monthly). Breast cancer **(c)** stands out from breast diseases **(a**, **b)** in oncological patients. In breast cancer, the statistical significance of the IgG response **(d)** results from the difference in the post-vaccination response separately for those vaccinated with Pfizer (BNT162b2) **(g)** and the administration of other vaccines **(i)**. All of them are working. Surprisingly, the response is more significant in unvaccinated patients **(f)** than in vaccinated patients **(e)** for breast cancer. In this group, the humoral response is significant for all three doses **(h, i)** of the vaccine used (S1, S2, and S3) regardless of the type (and combination of vaccines each other) **(j)**. It was observed **(k)**, regardless of vaccination (non-vaccinated group), a more significant increase in the humoral response in treated patients (radiotherapy, chemotherapy, and surgery) and a weaker post-vaccination response in groups of cancer patients over 60 years of age **(l)**. All of this contribute to the description of the breast cancer anti-infectious response to the COVID-19 pandemic, which may help to understand SARS-CoV-2 fight cancer. S0 (no vaccination), S1 (single vaccine: Johnson&Johnson(J&J), AstraZeneca (AZ), Moderna, Pfizer(COM; Comirnaty), S2A (full two-dose one vaccination) and S2B (two-dose vaccination Pfizer and Moderna), S3, S4, S5-additional vaccination. CHTH-chemotherapy; RTH-radiotherapy; SURG-surgery; n-amount of study samples. P-values represent comparison with groups (*) *p* < 0.05, (**) *p* < 0.001, (***) *p* < 0.0001

In breast cancer (Fig. 3), the response to vaccination was more noticeable by IgG levels for unvaccinated individuals (Fig. 3d, e, f), despite the effect of their treatment oncology (chemotherapy, radiotherapy, and surgery) (Fig. 3k) and the type and time vaccine administration (Fig. 3 , h). A higher share of antibodies was noticed in the population < 60 years old—breast cancer in vaccinated persons (Fig. 3l). BNT162b2 vaccine and other vaccines significantly (collectively as non-BNT162b2 due to small sample size) affected IgG antibody levels (Fig. 3i, j).

A noted natural feature of breast tissue is sensitivity to SARS-CoV-2 levels IgG for "non-inflammatory" breast (Fig. 3a) and "post-inflammatory" breast with cysts (Fig. 3 b) during monthly observation before the development of the cancer process (Fig. 3 c), and then its response to vaccination in 2021/2022 (Fig. 3 d).

### Lung and colon *cancer*

**Fig. 4:**
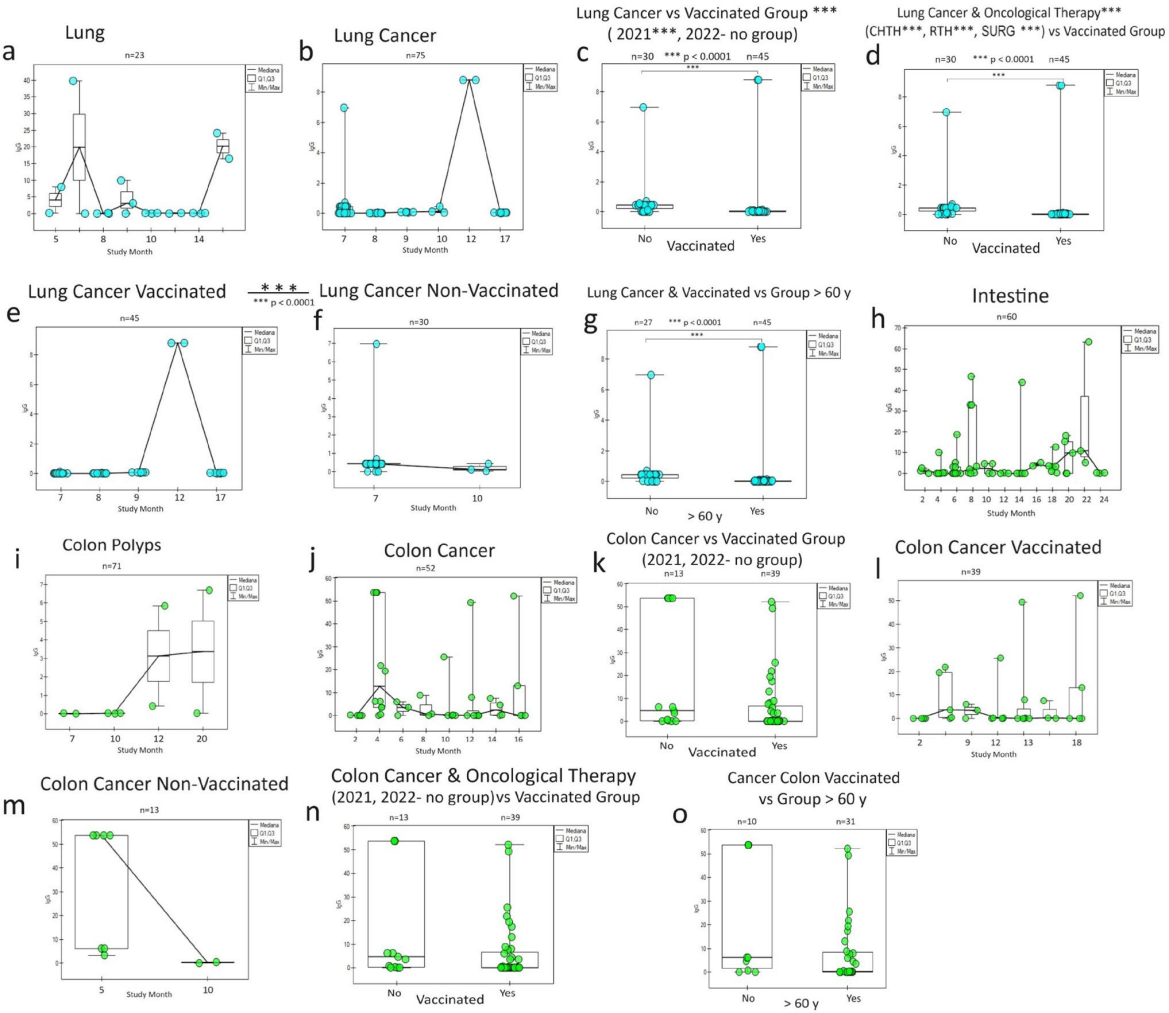
The graphs show differences in the humoral response of specific SARS-CoV-2 IgG in lung and colon cancer in response to COVID-19 vaccinations during a 2-year (monthly) follow-up. Both in the lungs **(a)** and in the intestine **(h, i)**, there are pre-oncological states that overlap with the tumour humoral response for the lungs **(b)** and large intestine **(j)**. Statistical correlations were found between the vaccinated group **(c, e, f)** and in the groups for oncological treatment **(d)** and the group of older people > 60 years **(g)** only in the case of lung cancer, but not in colon cancer **(k, l, m, n, o)**. In both cancers, there are no comparison groups to demonstrate the effects of the type of vaccines. It is noteworthy that colon cancer is significant despite similar relationships and the predominance of group unvaccinated cancer patients (as in breast cancer and lung cancer) over vaccinated ones **(c, k),** which also accounts for the specific humoral response of this cancer to SARS-CoV-2 infection. S0 (no vaccination), S1 (single vaccine: Johnson&Johnson(J&J), AstraZeneca (AZ), Moderna, Pfizer(COM; Comirnaty), S2A (full two-dose one vaccination), and S2B (two-dose vaccination Pfizer and Moderna), S3, S4, S5-additional vaccination. CHTH-chemotherapy; RTH-radiotherapy; SURG-surgery; n-amount of study samples. P-values represent comparison with groups (*) *p* < 0.0001

The humoral IgG response to vaccination in the lung cancer population (Fig. 4) is other than for breast cancer. As you can see, the reaction from the healthy tissue (Fig. 4a) is different from cancer's (Fig. 4 b). A similar response phenomenon again draws attention higher in unvaccinated patients (in subsequent years) than in vaccinated patients cancer patients (Fig. 4c, e, f) and in oncological patients (Fig. 4d). The lowest share of antibodies was noticed in the population > 60 years old—lung cancer in vaccinated persons (Fig. 4g).

Colon cancer (Fig. 4j): the only one from the gastrointestinal tract showed a more excellent IgG response than the other conditions (Fig. 4h, i) and the rest observed cancer (oesophagus, stomach, small intestine, and rectum). Even though the trend was there similar to lung cancer (higher share of non-vaccinated cancer antibodies patients), no statistically significant relationship was observed for colon cancer, both for those treated, vaccinated, and in age groups.

### Other cancers

Additionally, we observed a similar significant response in a group of vaccinated patients treated for kidney and testicular cancer (Table 2). In the entire group of vaccinated cancers, the response was different, but the strongest response was for breast cancer (Fig. 3), colon, and lung cancer (Fig. 4).

**Fig. 5:**
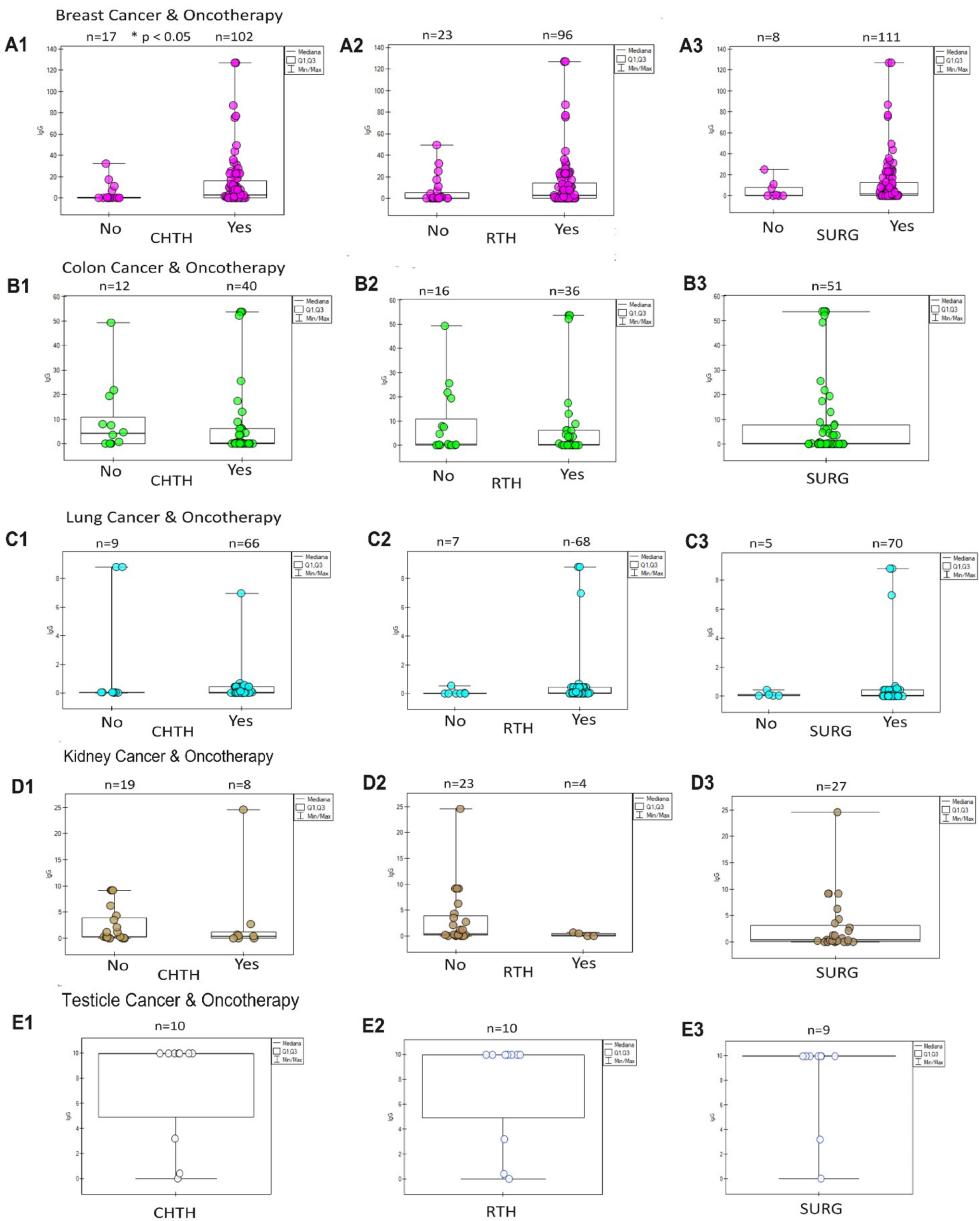
The diagrams show the humoral response of SARS-CoV-2 IgG antibodies to oncological therapy: chemotherapy (CHTH), radiotherapy (RTH), surgery (SURG) in oncological patients with breast cancer **(A1, A2, A3),** colon cancer **(B1, B2, B3)**, lungs **(C1, C2, C3)**, kidneys **(D1, D2, D3)**, testicles **(E1, E2, E3)**, states in which statistical significance was observed in the vaccinated and unvaccinated groups (Table 2). CHTH response is the strongest. Here, we observe a dual response to RTH: positive (rise IgG) in the breast, colon, and lung, and negative (fall IgG) in the kidney, and SURG response: positive (rise) in the breast, colon, and lung. When there is only one group in the study (testicle cancer and surgery in kidney cancer), the effect of oncotherapy is unknown. * The *p*-value is presented only in the CHTH breast cancer group—n-amount of study samples

The assessment of the response of tumours to oncological treatment is visible in Fig. 5. A typical positive response (increase in IgG) in the group of patients treated oncologically was observed in breast cancer (Fig. 5A1, A2, A3), intestinal cancer (Fig. 5B1, B2, B3), and lungs (Fig. 5C1, C2, C3). A negative response (decrease in IgG) was visible only in the group treated with CHTH (Fig. 5 D1) and RTH (Fig. 5 D2) of kidney cancer. The interpretation of treatment in small group kidney and testicular cancer is incomplete due to the lack of a control group (Fig. 5 D3, E1, E2, E3).

We also see the COVID-19-sensitive potential (positive) in non-vaccinated patients' non-oncological diseases, such as rheumatoid arthritis, Graves` disease, and fibroma (Table 2.).

The humoral response measured by the SARS-CoV-2 IgG and IgM antibody concentration naturally increased during the pandemic and after vaccination. It was higher among people < 60 years of age, which may be explained by the population's ageing of the immune system.

## Discussion

This study aimed to answer the question about the impact of the cancer process on the humoral response in oncological patients vaccinated against SARS-CoV-2 infection and in patients after COVID-19. The studies also drew attention to the potential impact of the anti-infective response, in this case, the antiviral response, on the potentiation of the anticancer response. These observations are important because a post-infectious or post-vaccination increase in the concentration of IgG antibodies, secondary response antibodies, provides a potential opportunity for many months of "strengthening" the humoral response, which is expected to have a significant impact on the course of anticancer therapy.

The type of cancer, its histopathological characteristics, and its stage of advancement generate differently intense and targeted immune responses [13]. The question, therefore, arises whether some cancers do not naturally generate a more excellent humoral response directed against the SARS-CoV-2. Recognized contraindications to the implementation of systemic cancer treatment include coexisting inflammation, systemic reinfections, herpes zoster, and herpes, leading to immunosuppression. On the other hand, a parallel viral infection, e.g. with the SARS-CoV-2 in oncological patients, due to the existing immune deficiency, may lead to the development and progression of an often life-threatening infectious process. In the context of the data cited, the question arises whether, despite these "alarm" signals, we should vaccinate oncology patients, including those undergoing anticancer therapy, with the virus.

The results contained in our previous work, which assessed the post-vaccination response to BNT162b2 (Pfizer: Comirnaty) and the reaction after COVID-19, based on a 2-year analysis of five cases of differentiated humoral response, showed surprising conclusions [14]. In the summary of the work, administering at least three doses of mRNA vaccine should serve as the basis for immunization, and a 3-month interval may be the best alternative to the vaccination schedule for non-immunocompromised (healthy) people. Our observations were confirmed in the works of other authors [6].

Given the above, the advisability of vaccinating people with the so-called risk groups, i.e. patients over 65 years of age with immunological deficiencies related to the ageing process, people with risk factors for the severe course of COVID-19, such as obesity, arterial hypertension, or circulatory failure.

Should we also vaccinate people with clinical signs of infection with SARS-CoV-2 despite the lack of confirmation of the presence of the virus by PCR tests, which, as we mentioned above, may be responsible for the low sensitivity of the tests used [15]? The magnitude of the post-vaccination response measured by the concentration of IgM and IgG antibodies varies individually; in the case of oncology patients, it is influenced by the nature of the tumour, its histopathological type, stage of advancement, and the therapy used [16]. In the case of breast cancer therapy based on CDK 4/6 inhibitors, which inhibit the cycle and division of cancer cells, it has a specific effect by inhibiting the effect of physiological estrogens on cancer cells [17]. The conducted research proved the safety of early immunization, i.e. 2 weeks after the end of COVID-19, in the population of women with breast cancer [18].

In the case of breast cancer chemotherapy, despite a rapid antibody response, a decrease in mainly S-RBD antibodies is usually observed [19]. In Canada, where COVID-19 vaccination coverage reached over 70% of the population, the incidence of breast and intestinal cancer decreased by almost half after the SARS-CoV-2 pandemic [19]. It is suggested that 10–20% of the general population may not show an antibody response for various reasons, while almost 80% of oncology patients experience infections asymptomatically or with few symptoms [20].

## Summary

Preliminary results of the studies that are the subject of this study show significant variability of the humoral response in patients followed for 2 years. In our opinion, the most sensitive and correlating with the clinical condition of the assessed patients are specific SARS-CoV-2 in the IgG class, the significant increase of which was observed in the case of reinforcement with "subsequent" doses of the vaccine [21]. We used the criteria for diagnosing virus activation in asymptomatic people proposed in the study of post-vaccination immunity of our patients in 2021, adopting a cutoff for SARS-CoV-2 at the level of IgG > 0.2 AU/ml and IgM > 1.0 AU/ml [22], which significantly improved detection of seroconversion of the SARS-CoV-2 in the population patients undergoing oncological treatment. The observations contained in this study prove that vaccination of oncological patients against SARS-CoV-2 infection may be a potential target in multifactorial therapy of cancer diseases [23, 24].

## Conclusions

We observed a much greater COVID-19-dependent response (concentrations IgG and IgM SARS-CoV-2) in cancer breast, lung, and colon patients, independent of oncological therapy and vaccination (Figs. 3, 4).

An argument for revaccination in this group of people immunocompetent, with an apparent deficit of haemopoiesis after chemotherapy or radiotherapy, is that their early (primary) immune response is naturally weak by a small amount of the first specific SARS-CoV-2 IgG antibodies determined in research.

Additionally, we observed an ageing effect on the immune system in people 60 + of both sexes, despite the population increase in humoral response in groups aged during 2 years of observation.

## Data Availability

No datasets were generated or analysed during the current study.
